# Claudin-18 status and its correlation with HER2 and PD-L1 expression in gastric cancer with peritoneal dissemination

**DOI:** 10.1007/s10120-024-01505-6

**Published:** 2024-05-09

**Authors:** Haruki Ogawa, Hiroyuki Abe, Koichi Yagi, Yasuyuki Seto, Tetsuo Ushiku

**Affiliations:** 1 Department of Pathology, Graduate School of Medicine, the University of Tokyo, 7-3-1 Hongo, Bunkyo-Ku, Tokyo, 113-0033 Japan; 2 Department of Gastrointestinal Surgery, Graduate School of Medicine, the University of Tokyo, 7-3-1 Hongo, Bunkyo-Ku, Tokyo, 113-0033 Japan

**Keywords:** Gastric cancer, Claudin-18, Peritoneal dissemination, Molecular targeted therapy, Immunohistochemistry

## Abstract

**Background:**

Gastric cancer with peritoneal dissemination (PD) has a dismal prognosis, and current treatments have shown little efficacy. CLDN18.2-targeted therapies have shown promising efficacy against gastric cancers that express high levels of CLDN18. Because of the limited information regarding CLDN18.2 status in PD, we analyzed PD-positive gastric cancers for CLDN18 status in both primary and PD, along with HER2 and PD-L1 combined positive score (CPS).

**Methods:**

Immunohistochemical analyses were performed on 84 gastric cancer cases using paired primary and PD tissue samples.

**Results:**

At 40% cut-off, CLDN18 was positive in 57% (48/84) primary tumors and in 44% (37/84) PDs. At 75% cut-off, 28.6% (24/84) primary tumors and 20.2% (17/84) PDs were CLDN18-positive. The concordance rate between primary tumors and PD was 79.8% at 40% cut-off and 75% at 75% cut-off. When comparing biopsy and surgical specimens, the concordance rates were 87.5% at 40% cut-off and 81.3% at 75% cut-off. Within a tumor, the superficial area tended to have a higher CLDN18-positive rate than the invasive front (*P* = 0.001). Although HER2 -positivity was only 11.9% in this cohort, CLDN18 positivity in HER2-negative tumors (n = 74) was relatively high: 60.8% at 40% cut-off and 28.4% at 75% cut-off. Among double-negative (HER2 − and PD-L1 CPS < 1) tumors, CLDN18 positivity was 67.6% at 40% cut-off and 26.5% at 75% cut-off.

**Conclusions:**

CLDN18 expression is generally maintained in PD and is relatively high even in double-negative tumors, making it a promising therapeutic target for PD-positive gastric cancer.

**Supplementary Information:**

The online version contains supplementary material available at 10.1007/s10120-024-01505-6.

## Introduction

Gastric cancer is the fourth leading cause of cancer-related deaths worldwide [1]. The morbidity and mortality rates are declining because of the decreasing prevalence of *Helicobacter pylori* infection, widespread *H.pylori* eradication therapy, and early detection through endoscopy. However, the prognosis of patients with advanced gastric cancer, especially those with peritoneal dissemination (PD), is still poor [2]. Chemotherapy, anti-human epidermal growth factor receptor 2 (HER2) antibodies (such as trastuzumab), anti-VEGFR2 antibodies (such as ramucirumab), and immune checkpoint inhibitors are standard therapies for unresectable or recurrent gastric cancer. PD-positive gastric cancers typically exhibit a diffuse-type histology and this subtype is HER2-negative in most cases. PD-L1expression is also relatively low in the diffuse-type subtype when compared to the microsatellite instability and Epstein-Barr virus-positive subtypes. Therefore, new treatment strategies for PD-positive gastric cancers are urgently required.

Recently, zolbetuximab, which targets the tight junction molecule claudin-18 isoform 2 (CLDN18.2), was developed. Zolbetuximab is a monoclonal immunoglobulin G1 (IgG1) antibody that binds CLDN18.2 and induces antibody- and complement-dependent cellular cytotoxicity. Randomized phase 2 (FAST, NCT01630083) and phase 3 trials (SPOTLIGHT, NCT03504397, and GLOW, NCT03653507) have demonstrated the clinical efficacy of zolbetuximab in combination with chemotherapy in patients with HER2-negative unresectable/recurrent gastric cancer showing high expression of CLDN18.2 [3–5]. In these clinical trials, CLDN18.2 expression was assessed using immunohistochemistry with an anti-CLDN18 antibody (clone 43-14A), and the proportion of neoplastic cells with moderate (2 +) or strong (3 +) expression was evaluated at different cut-off values. In the FAST trial, a cut-off value of 40% was used [5], whereas a cut-off value of 75% was used in the SPOTLIGHT and GLOW trials [3, 4]. Although the clone 43-14A antibody reacts with both CLDN18.1 and CLDN18.2, the antibody was used for the detection of CLDN18.2 in these clinical trials because CLDN18 expressed in the stomach is exclusively CLDN18.2, not CLDN18.1 [6, 7]. Therefore, this antibody was used to analyze CLDN18.2 expression in the present study.

Earlier studies have suggested that CLDN18.2 expression decreases during cancer progression, but information regarding the concordance between primary and metastatic lesions is still limited [8, 9]. Rohde et al. reported that CLDN18.2 expression status in primary gastric cancers was frequently maintained in regional lymph node metastases [10]. Regarding PD, a recent study analyzed CLDN18 expression using effusion cell block samples, demonstrating a high concordance (83.7%) between primary tumor tissue and effusion cells [11]. However, no study has evaluated CLDN18 expression using tissue samples, not suspended cells in ascites, that was resected from PD. Because of its clinical importance and the lack of data, we examined the expression of CLDN18 in both primary tumors and PD to determine the applicability of zolbetuximab in patients with PD-positive gastric cancer. We also focused on intratumoral heterogeneity by comparing the CLDN18 status among different tumor areas (surface vs. center vs. invasive front) and different sample types (biopsy vs. surgical resection). In addition, the correlation between the CLDN18 status and other biomarkers, including HER2 and PD-L1, was clarified.

## Materials and methods

### Tissue samples

**Fig. 1:**
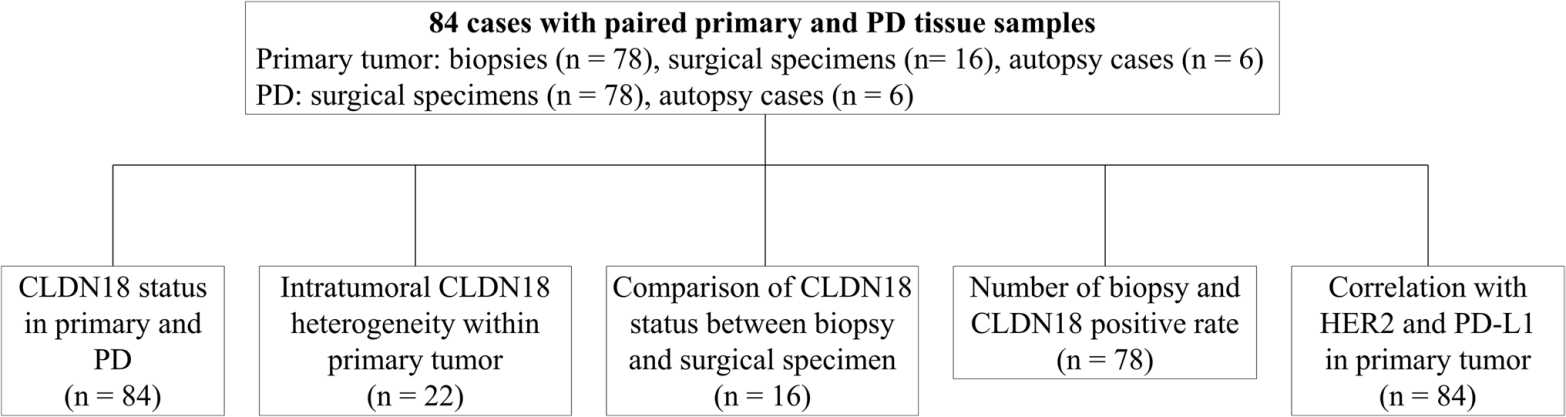
Summary of specimens used in each analysis. A total of 84 cases with gastric cancer with PD were included in the study, and five main analyses were performed. The numbers in parentheses indicate the number of cases studied

This retrospective study included 84 gastric cancer cases with tissue samples from both primary and PD, collected from the pathological archive at The University of Tokyo Hospital between April 2000 and December 2021. For primary lesions, the tissue types included 78 biopsies, 16 surgical specimens, and six autopsy cases, and PD tissues includes 87 surgical specimens and six autopsy cases (Fig. 1). In nine cases, both pre- and post-chemotherapy samples were available. Clinicopathological data, including age, sex, tumor location, tumor size, T stage, lymphatic invasion, and venous invasion were collected from pathological records. Gastric cancer histology was classified according to the Lauren classification [12].

Follow-up data were collected from the medical record. Four patients who were revealed to have gastric cancer for the first time at autopsy were excluded from survival analyses. Overall survival (OS) time was defined as period from the date of diagnosis to the date of death of any cause or last follow-up. Because patients with PD could not usually receive curative resection, recurrence free survival analysis was not performed.

This study was performed according to the Declaration of Helsinki, and the study protocol was approved by the institutional review board (approval number G3521).

### Immunohistochemistry

Sections of 3-μm thickness were prepared from formalin-fixed paraffin-embedded tissue blocks. Immunohistochemistry was performed using a Ventana Benchmark Ultra automated immunostainer (Ventana Medical Systems, Tucson, AZ, USA), according to the manufacturer’s instructions. Immunohistochemistry of CLDN18 was performed using the mouse monoclonal antibody clone 43-14A (Ventana Medical Systems).

Membrane expression of CLDN18 was evaluated based on the staining intensity and proportion. The staining intensity was classified as 0 (no staining), 1 + (weak staining), 2 + (moderate staining), and 3 + (strong staining). In this study, CLDN18 positivity was defined as moderate (2 +) or strong (3 +) CLDN18 staining using two cut-off values: ≥ 40% (FAST trial eligibility criterion) and ≥ 75% (SPOTLIGHT and GLOW trial eligibility criteria). In specimens resected by surgery or autopsy, the primary tumor area was subdivided into three areas (superficial, central, and invasive front), and CLDN18 expression was evaluated separately in each area and in the whole tumor. CLDN18 expression in normal gastric epithelium served as an internal positive control. Immunohistochemical evaluations were performed independently by two observers (H.O. and H.A.) who were blinded to the clinicopathological data. In case of disagreement, the slides were re-evaluated by the two observers to reach a final decision.

HER2 status was evaluated based on the American Society of Clinical Oncology guidelines [13]. In brief, immunohistochemistry of HER2 was performed using a rabbit monoclonal anti-HER2 antibody (clone 4B5, prediluted, Roche, Basel, Switzerland) and evaluated at a score of 0, 1 + , 2 + , or 3 + . For cases with a score of 2 + , dual-color ISH (DISH) for HER2 was performed using the Ventana INFORM HER2 DualColor ISH Kit (Roche) to assess HER2 amplification. Cases with immunohistochemistry 3 + or 2 + with *HER2* amplification were considered HER2-positive.

Immunohistochemistry for PD-L1 was performed using a rabbit monoclonal anti-PD-L1 antibody (clone SP263, prediluted; Roche). PD-L1 expression was evaluated using the combined positive score (CPS) [14]. The PD-L1 CPS was defined as the number of PD-L1-expressing cells (tumor cells, lymphocytes, and macrophages) divided by the total number of tumor cells, multiplied by 100. PD-L1 expression was classified as CPS < 1, 1 ≤ CPS < 5, and CPS ≥ 5 based on the criterion of the preceding clinical trial for nivolumab treatment for patients with gastric cancer [15]. In this study, HER2 and PD-L1 were evaluated only in the primary tumors.

### Statistical analyses

All statistical analyses were performed using the JMP Pro 17 software (SAS Institute Inc., Cary, NC, USA). Continuous variables were analyzed using a paired Student’s t-test, and categorical data were analyzed using Fisher’s exact test. The Kaplan–Meier method was used to draw survival curves and estimate OS. OS was compared between CLDN18-positive and -negative groups by Wilcoxon test. The Cox proportional hazards model was used to calculate the hazard ratio (HR) and 95% confidence interval (CI). Statistical significance was set at P < 0.05.

## Results

### CLDN18 status in primary and its clinicopathological correlation

**Fig. 2:**
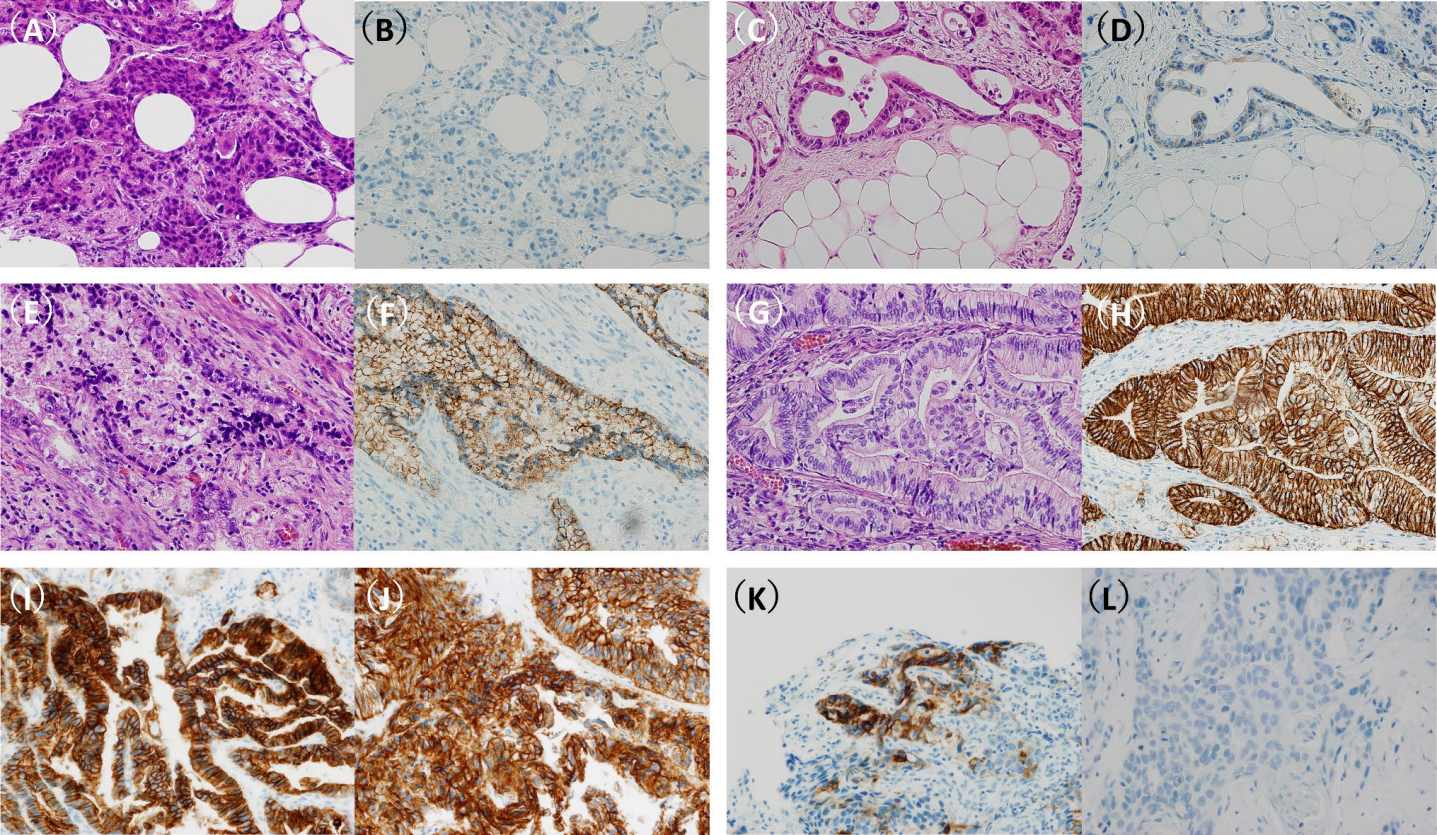
Representative images of CLDN18 immunohistochemistry. Pair images (H&E staining and CLDN18 immunohistochemistry) of four categories in CLDN18 intensity: 0, no expression (**A and B**); 1 + , weak expression (**C and D**); 2 + , moderate expression (**E and F**); and 3 + , strong expression (**G and H**). CLDN18 immunostainings of a representative case with CLDN18-positive in both the primary (**I**) and peritoneal dissemination (**J**). CLDN18 immunostainings of a case with discordant results that showed CLDN18-positive in the primary tumor (**K**) but negative in the peritoneal dissemination (**L**)

Figure 2 shows representative images of each CLDN18 immunohistochemistry score. Among primary tumors (n = 84), 48 (57.1%) showed ≥ 40% and 24 (28.6%) showed ≥ 75% CLDN18 expression (Table 1). There was no significant correlation between the CLDN18 status and sex, age, or tumor site. Histologically, 66 primary tumors (78.6%) were of the diffuse type and 18 (21.4%) were of the intestinal type. There was no significant difference in CLDN18 status between intestinal- and diffuse-type histology.

**Table 1:** CLDN18 status at two cut-off values and clinicopathological characteristics in primary tumors and peritoneal disseminations

	n	CLDN18 in primary tumors						CLDN18 in peritoneal disseminations					
		40% cut-off			75% cut-off			40% cut-off			75% cut-off		
		−	+	P-value^*^	−	+	P-value^*^	−	+	P-value^*^	−	+	P-value*
Total	84	36	48		60	24		47	37		67	17	
Sex				0.35			0.62			0.82			0.78
Male	55	26	29		38	17		30	25		43	12	
Female	29	10	19		22	7		17	12		24	5	
Age				0.27			0.48			0.13			0.60
< 65	44	16	28		33	11		21	23		34	10	
≥ 65	40	20	20		27	13		26	14		33	7	
Tumor site				0.53			0.09			0.06			0.08
U	17	7	10		9	8		7	10		10	7	
M	51	20	31		37	14		27	24		43	8	
L	16	9	7		14	2		13	3		14	2	
Histologic type				0.11			0.77			1.0			1.0
intestinal	18	11	7		12	6		10	8		15	3	
diffuse	66	25	41		48	18		37	29		52	14	

*CLDN* Claudin

^*^Fisher’s exact test

In overall survival analysis, no significant difference was observed between CLDN18-positive and –negative groups at either 40% or 75% cut-offs (Supplementary Fig. 1). Comparing CLDN18 status in PD before and after chemotherapy in nine patients, CLDN18 expression was positive in four (44.4%) (40% cut-off) and two (22.2%) (75% cut-off) patients before chemotherapy, whereas it was positive in five (55.6%) (40% cut-off) and four (44.4%) (75% cut-off) patients after chemotherapy. The concordance rate of CLDN18 status before and after chemotherapy was 88.9% (8/9) at 40% cut-off and 66.6% (6/9) at 75% cut-off.

### Comparison of CLDN18 expression between primary and PD

**Fig. 3:**
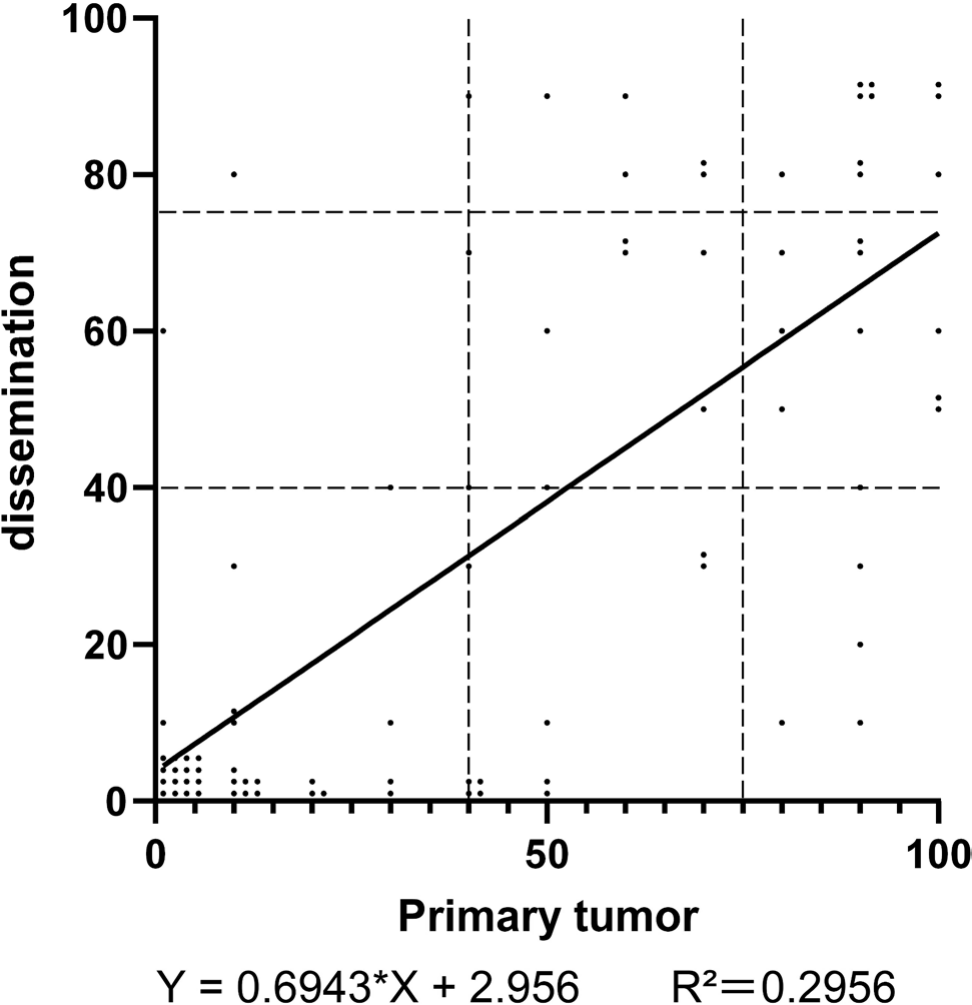
Comparison of CLDN18 expression between primary tumors and peritoneal dissemination. The percentage of CLDN18-positive tumor cells in the primary tumor is shown on the X-axis, and the percentage of peritoneal dissemination is shown on the Y-axis. The line represents a linear regression curve

In PD (n = 84), 37 (44.0%) showed ≥ 40% and 17 (20.2%) showed ≥ 75% CLDN18 expression. The relationship between primary tumors and PD is shown in Fig. 3 and Tables 2. Although a positive correlation existed between CLDN18 expression in primary tumors and PD, CLDN18 expression in PD was significantly lower than that in primary tumors (P < 0.001). The concordance rate between primary tumors and PD was 79.8% (67/84) at the 40% cut-off and 75% (63/84) at the 75% cut-off.

**Table 2:** CLDN18 expression in primary tumors and peritoneal disseminations

(A)			
40% cut-off		CLDN18 in peritoneal disseminations	
		−	+
CLDN18 in primary tumors	−	33 (39.3%)	3 (3.6%)
	+	14 (16.7%)	34 (40.5%)
concordance rate 79.8%		P* < 0.001	

(B)			
75% cut-off		CLDN18 in peritoneal disseminations	
		−	+
CLDN18 in primary tumors	−	53 (63.1%)	7 (8.3%)
	+	14 (16.7%)	10 (11.9%)
concordance rate 75.0%		P^*^ = 0.005	

*CLDN* Claudin

^*^Fisher’s exact test

### Intratumoral CLDN18 heterogeneity

**Fig. 4:**
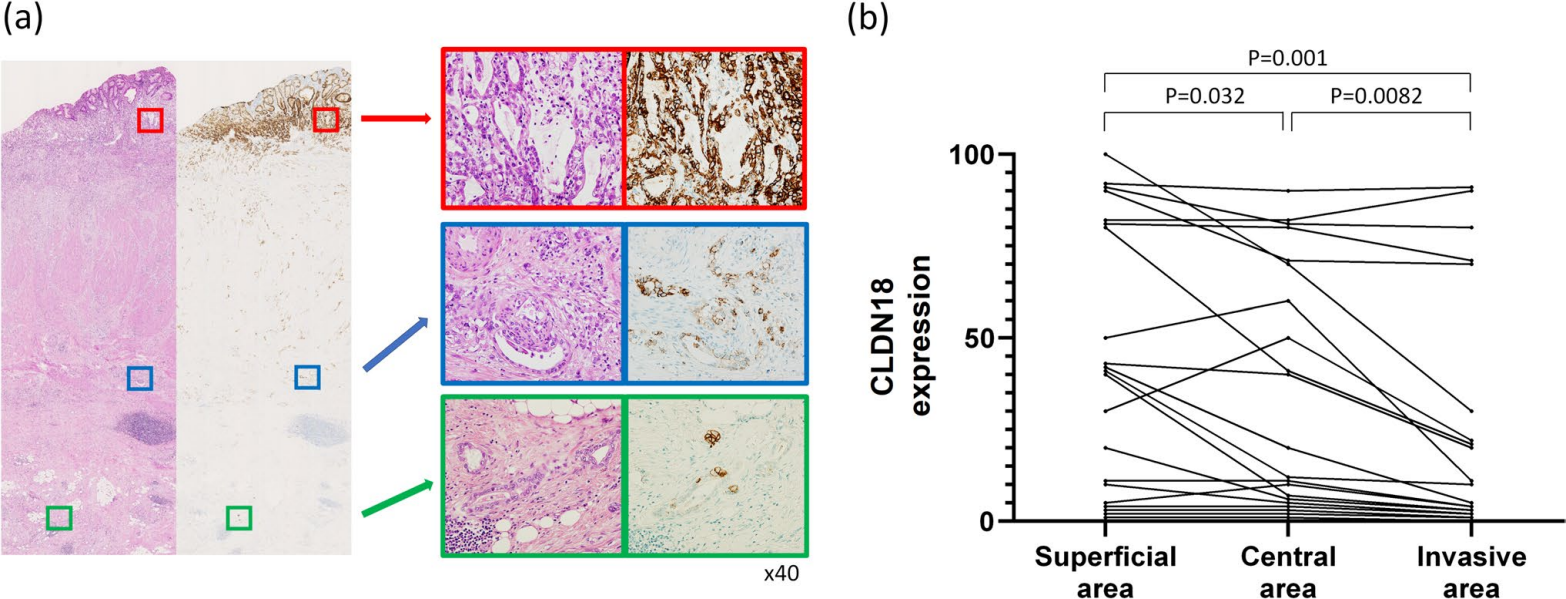
Intratumoral CLDN18 heterogeneity. **a** A representative case exhibiting heterogeneous CLDN18 expression. Loupe view of H&E and CLDN18 immunostaining (left). At higher power magnification, on the right, diffuse CLDN18 expression in the superficial area (upper pictures in the red box), partial positive staining in the center (middle pictures in the blue box), and almost negative staining in the invasive front (lower pictures in the green box) were noted. **b** The Percentage of CLDN18-positive neoplastic cells in the superficial, central, and invasive front areas. CLDN18 expression tended to decrease from the superficial to deep areas

The intratumoral heterogeneity of CLDN18 expression was examined in 22 primary tumors that were surgically resected or autopsied. CLDN18 expression was evaluated separately in the superficial, central, and invasive frontal areas (Fig. 4a). The positivity rate of CLDN18 was highest in the superficial areas, lower in the central areas, and lowest in the invasive front areas (superficial vs. central, *P* = 0.032; central vs. invasive front, *P* = 0.008; superficial vs. invasive front, *P* = 0.001) (Fig. 4b).

### Comparison between biopsies and surgical specimens

CLDN18 expression was compared in 16 cases in which both biopsy and surgical specimens of the primary tumor were available (Supplementary Fig. 2). No significant difference were elucidated between the biopsy and resected specimens (P = 0.44), with the concordance rate between the biopsy and resection specimens being 87.5% (14/16) at a 40% cut-off and 81.3% (13/16) at a 75% cut-off.

### Number of biopsies and CLDN18 positive rate

To determine whether the positivity of CLDN18 depends on the number of tissue fragments obtained by biopsy, we examined the relationship between the number of fragments containing tumor tissue and CLDN18 positivity in 78 cases for which biopsy samples were available. In this cohort, the number of biopsied fragments, including tumor tissue, was one in 11 cases, two in 21 cases, three in 20 cases, four in 14 cases, five in eight cases, and six in four cases, with an average of 3.0 fragments per biopsy. At a cut-off of 40%, the CLDN18-positive ratio was 65.4% (34/52) of the ≤ 3 biopsies group and 46.2% (12/26) of the ≥ 4 biopsies group. At a cut-off of 75%, it was 32.7% (17/52) of the ≤ 3 biopsies group and 26.9% (7/26) of the ≥ 4 biopsies group. Therefore, no significant difference was observed between the number of biopsied fragments and CLDN18 positive ratio.

### Correlation with HER2 and PD-L1

**Fig. 5:**
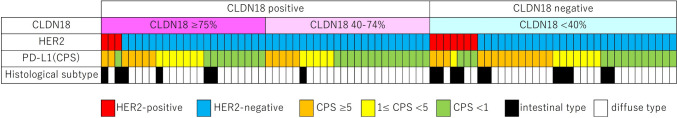
Correlation between CLDN18 status and HER2, PD-L1 CPS, and Lauren histological classification. Although CLDN18 status was not significantly correlated with HER2 and PD-L1, a significant proportion of HER2-negative or PD-L1 CPS < 1 cases were CLDN18-positive

The expression profiles of HER2 and PD-L1 (CPS) as well as CLDN18 status are shown in Fig. 5. HER2-test revealed that 11.9% (10/84) of the primary tumors were HER2-positive, including 33.3% (6/18) of intestinal-type and 6.7% (4/66) of diffuse-type histology. PD-L1 CPS was ≥ 5 in 31.0% (26/84), 1–5 in 23.8 (20/84), and < 1 in 45.2% (38/84) cases.

No significant correlations were noted between CLDN18 and HER2, or between CLDN18 and PD-L1 CPS in the primary tumors. CLDN18-positivity in HER2-negative tumors (n = 74) was 60.8% (45/74) at 40% cut-off and 28.4% (21/74) at 75% cut-off. Among double-negative (that is, HER2 − and PD-L1 CPS < 1) tumors, CLDN18 positivity was 67.6% at 40% cut-off and 26.5% at 75% cut-off.

## Discussion

The present study demonstrated that CLDN18 positivity in PD-positive gastric cancers was 54.8% at 40% cut-off and 28.6% at 75% cut-off in primary tumors, and the frequency was slightly lower in disseminated lesions, showing positivity in 44% at 40% cut-off and 20.2% at 75% cut-off. In addition, we showed intratumoral CLDN18 heterogeneity with a tendency to decrease from the superficial to the deep side of the primary tumor, which is in line with earlier publications [8, 16]. Because CLDN18 is a tight junction protein, the loss of CLDN18 may induce epithelial-mesenchymal transition and promote cancer cell dissemination into the peritoneal cavity. Interestingly, studies have reported that CLDN18 expression is relatively preserved in lymph node metastases [10, 17], suggesting that the significance of the loss of CLDN18 expression is not as high as that in PD. Although there was a tendency for reduced CLDN18 expression in PD, the concordance rate of CLDN18 status between primary and PD tumors was more than 75%, suggesting that zolbetuximab may be effective for patients with PD, even when tested for CLDN18 status in primary tumors. However, because a small subset of cases showed highly discordant CLDN18 expression between primary tumors and PD, it is preferable to test CLDN18 expression in PD as well as in primary tumors when considering the indication of zolbetuximab.

Regarding biomarker testing, studies have repeatedly reported the significance of the types of specimens tested (that is, biopsy vs. surgical resection) and the number of biopsy fragments, especially in the setting of HER2-testing because of the heterogeneous pattern of HER2 expression [18–22]. Based on our observations, the CLDN18 expression pattern is also usually heterogeneous, similar to HER2. In our analyses, no significant correlation was noted between CLDN18 positivity and the type of specimen (biopsies vs. surgical specimens) or the number of biopsy fragments. The reason for this finding is probably the higher CLDN18 positivity in the superficial area from which biopsy is taken. However, in several cases, there was a mismatch in CLDN18 positivity between the biopsy and surgical specimens, such as CLDN18-negative biopsy and CLDN18-positive paired surgical specimens. Owing to the limited number of specimens analyzed in this study, further studies are required to determine the optimal biopsy procedure for assessing CLDN18 expression in gastric cancer.

Earlier studies, as well as our current study, demonstrated that there was no significant difference in CLDN18 expression between HER2-positive and -negative cases [17, 23, 24]. Among HER2-negative cases (n = 74) in our cohort, CLDN18 was positive in 45 cases (60.8%) at the 40% cut-off and 21 cases (28.4%) at the 75% cut-off. As HER2 is more frequently positive in the intestinal type than in the diffuse type [25], gastric cancer with PD, which is predominantly the diffuse type, is usually not a target of anti-HER2 drugs. In contrast, CLDN18 expression was frequently observed in the diffuse and intestinal types. Thus, zolbetuximab is a promising drug for patients with HER2-negative gastric cancer for whom anti-HER2 drugs are not indicated.

In a previous study, CLDN18 expression was significantly lower in gastric cancer with PD than those without PD [26]. This is consistent with our result that CLDN18-positive ratio in this study was lower than those in the FAST and SPOTLIGHT studies. Also, PD-L1 expression has been reported to be lower in diffuse-type than intestinal-type [15, 27] and in distant metastasis than primary tumor [28, 29]. Considering the relatively low PD-L1 expression and moderate CLDN18 positivity regardless of PD-L1 status, CLDN18 is a promising therapeutic target for patients with PD-positive gastric cancer who exhibit low PD-L1 expression. Among double-negative (HER2 − and PD-L1 CPS < 1) tumors, CLDN18-positivity was 67.6% at 40% cut-off and 26.5% at 75% cut-off. Therefore, zolbetuximab is expected to partially meet the unmet need for the treatment of PD-positive gastric cancer.

The present study had several limitations. First, the study was retrospectively designed in a single institution, and the number of cases was relatively small because PD is infrequently surgically resected in routine clinical practice for gastric cancer treatment. However, the present study is the first to examine CLDN18 expression in resected PD tissues. Although a recent study examined CLDN18 expression in ascites cytology specimens, our study is the first to evaluate PD nodules because the pharmacokinetics, including the presence of blood supply, are notably different between floating cells in ascites and lesions that form disseminated nodules in the peritoneum. This is an important point because zolbetuximab is administered intravenously and primarily targets peritoneal invasive cancer tissues with blood supply, and therefore information concerning CLDN18 status in the resected PD tissue samples, not just tumor cells floating in ascites, is needed. Second, we used clone SP263 to detect PD-L1 expression, which is different from companion diagnostic PD-L1 testing for gastric cancer, the 22C3 pharmDx assay (Dako, Santa Clara, CA, USA) or the 28–8 pharmDx assay (Dako) [14, 30]. However, previous studies have reported that 22C3 and SP263 antibodies are highly concordant in PD-L1 scoring. Thus, SP263 is considered equivalent to these companion diagnostic methods [31–33]. Finally, HER2 and PD-L1 were evaluated only in the primary tumors in this study. Although the information is still limited, the concordance ratio between primary and PD tissues was reported as 73.1% in HER2 status [34]. Concerning PD-L1 status, the concordance ratio between primary and distant metastasis (metastatic sites are not specified) was reported as 69.4% [35]. From these observations, it appears that the concordance rates between primary and metastatic sites for the three markers seem around 70%.

In conclusion, to the best of our knowledge, this is the first study to compare CLDN18 expression between primary tumors and PDs. Although CLDN18 expression tended to be mildly lower in deeper than in superficial areas, and also in disseminated lesions, the concordance rate of CLDN18 positivity between primary tumors and PDs was more than 75%. Our findings suggest that zolbetuximab is a promising treatment option for patients with PD-positive gastric cancer.

### Supplementary Information

Below is the link to the electronic supplementary material.

Supplementary file1 (DOCX 359 KB)
