# Supplementary material for: Claudin-18 status and its correlation with HER2 and PD-L1 expression in gastric cancer with peritoneal dissemination
Source: Gastric Cancer. 2024 May 9;27(4):802–10. doi: 10.1007/s10120-024-01505-6 (PMC11193835; doi:10.1007/s10120-024-01505-6)

**Claudin-18 status and its correlation with HER2 and PD-L1 expression in gastric cancer with peritoneal dissemination**

Haruki Ogawa M.D.,^1,2^ Hiroyuki Abe M.D., Ph.D.,^1^ Koichi Yagi M.D., Ph.D.,^2^ Yasuyuki Seto, M.D., Ph.D.^2^ & Tetsuo Ushiku M.D., Ph.D.^1^

1 Department of Pathology, Graduate School of Medicine, the University of Tokyo, 7-3-1 Hongo, Bunkyo-ku, Tokyo 113-0033, Japan.

2 Department of Gastrointestinal Surgery, Graduate School of Medicine, the University of Tokyo, 7-3-1 Hongo, Bunkyo-ku, Tokyo 113-0033, Japan.

H.O. and H.A. equally contributed to this work.

**Correspondence**:

Tetsuo Ushiku, MD, PhD

Department of Pathology, Graduate School of Medicine, The University of Tokyo

Tel: 81-3-5841-3341　　E-mail: [usikut@g.ecc.u-tokyo.ac.jp](mailto:usikut@g.ecc.u-tokyo.ac.jp)

Article type: original article

Submitted to Journal: Gastric Cancer

**Supplementary Figure 1. Correlation between CLDN18 status and overall survival.**

1. 40% cut-off. (b) 75% cut-off.


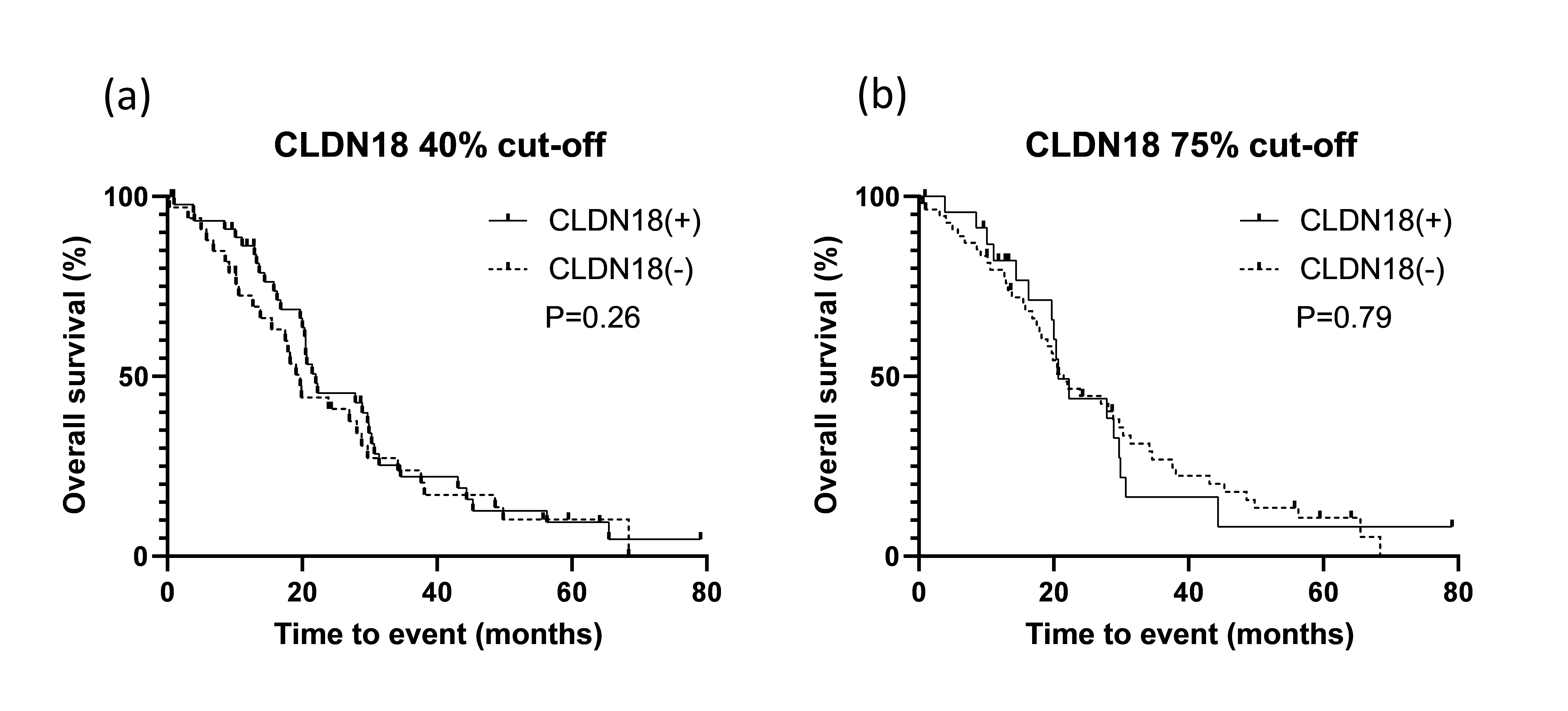


**Supplementary Figure 2. Comparison of CLDN18 expression between biopsy and surgical specimens**

There was no significant difference between the biopsy and surgical specimens, although several examples show discrepancies across the cut-offs.


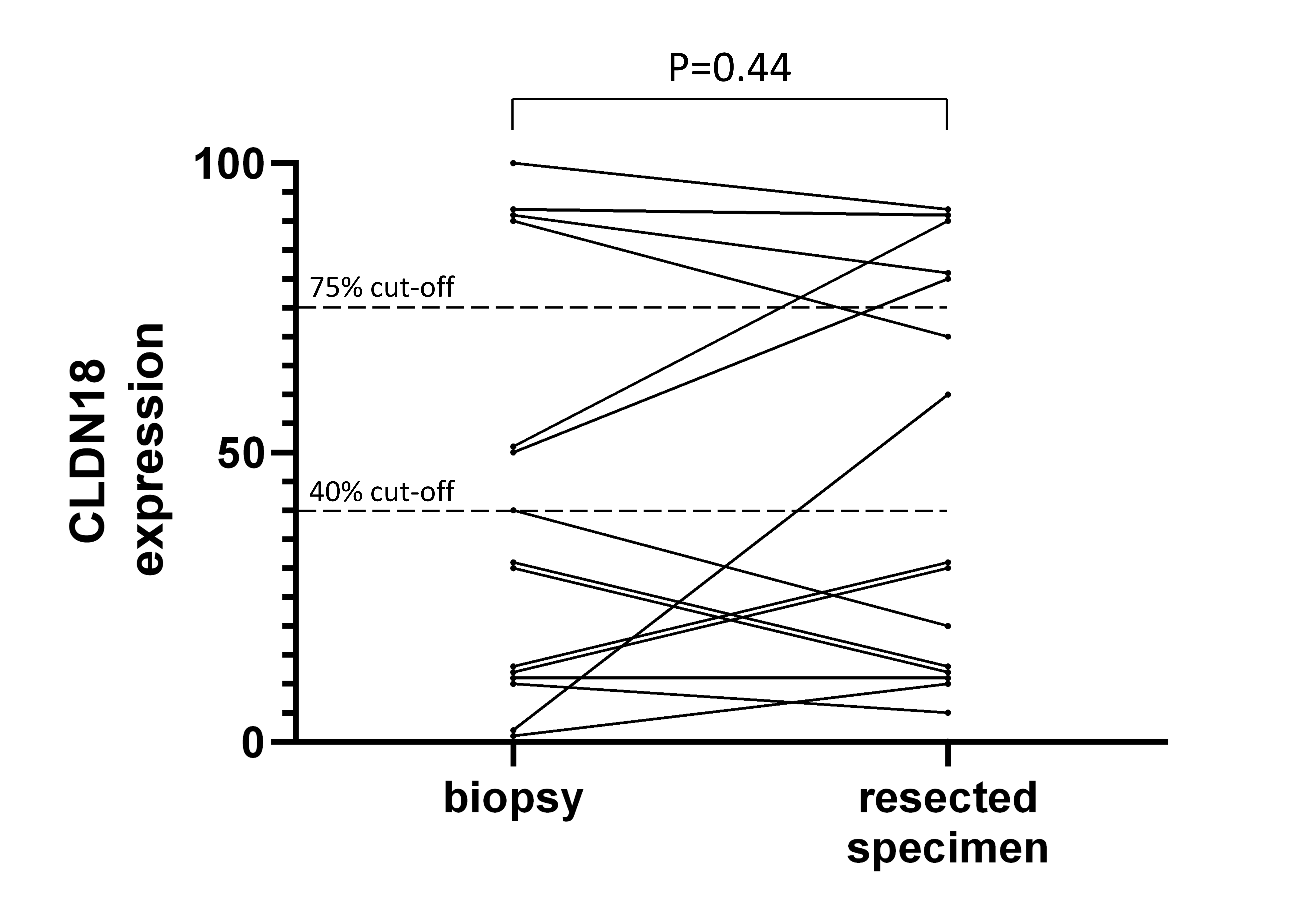

Supplement: Supplementary file 1 — Supplementary file1 (DOCX 359 KB) [file 10120_2024_1505_MOESM1_ESM.docx]
